# Do international standards for hygienic handrubs reflect realistic usage?

**DOI:** 10.1186/2047-2994-4-S1-P305

**Published:** 2015-06-16

**Authors:** MAC Wilkinson, C Bradley, J Hines, K Ormandy, A Fraise

**Affiliations:** 1 Hospital Infection Research Laboratory, Queen Elizabeth Hospital Birmingham, Birmingham, UK; 2 Deb Research and Development, Deb Group Ltd., Denby, UK

## Introduction

Hygienic handrubs are widely used in healthcare to reduce the probability of pathogens being spread via the hands. International standards such as EN 1500[1] have been developed to ascertain the efficacy of handrubs claiming antimicrobial action. This standard compares a reference product (2 x 3ml applications of 60% v/v isopropanol (IPA) over 60 seconds) with a test product(s). However, as WHO guidelines[2] recommend that drying times are limited to 30 seconds, does the standard represent realistic usage?

## Objectives

This study compares the volumes of alcohol-based hand rub (ABHR) applied with drying time, antimicrobial efficacy and user acceptability. The ABHR’s studied are 60% v/v IPA and the two WHO – recommended handrub formulations, i.e. 80% v/v ethanol and 75% v/v IPA, both of which contain 1.45% v/v glycerol.

## Methods

Fifteen volunteers were recruited to test six volumes of the three ABHR’s, ranging from 0.5ml to 3ml. The drying time and user acceptability were recorded. Five volunteers were then selected to test the same volumes in the manner of an EN 1500 test. The log_10_ reduction factor (RF) in *E. coli* K12 was calculated, in addition to whether the volunteers’ hands were dry at the end of the procedure.

## Results

Both RF and drying time significantly increase as the volume of ABHR increases (*p*<0.05; Friedman test). The user comments also displayed a significant relationship with volume (*p*<0.05, ξ^2^ test), with the majority of comments negative at 3ml. In the EN 1500 – style test, only the two smallest volumes dried fully for all ABHR’s (p<0.05, ξ^2^ test).

## Conclusion

EN1500 was not originally designed as a means to define product dosage; however this is increasingly becoming normal practice. Our data reinforce the need for standards to more accurately reflect the volumes of handrubs that are used in practice. One possible solution involves two – tiered standards, that test both high volume / long drying times and low volume / short drying times, thus allowing formulations to be tested against a reference product under both ideal and realistic conditions.

## Disclosure of interest

None declared.
